# Associations between household solid fuel use, kitchen ventilation, and depressive symptoms among older adults in China: a national cross-sectional study

**DOI:** 10.3389/fpubh.2026.1920233

**Published:** 2026-09-01

**Authors:** Tongxu Zhang, Chen Wu, Yuhan Liu, Jingshuang Qin, Xiaojie Pan, Na An, Min Li

**Affiliations:** 1 Department of Endocrinology and Metabolism, The First Hospital of China Medical University, Shenyang, Liaoning, China; 2 School of Nursing, China Medical University, Shenyang, Liaoning, China

**Keywords:** Chinese longitudinal healthy longevity survey, cross-sectional study, depressive symptoms, household solid fuel use, kitchen ventilation, older adults

## Abstract

**Objective:**

Household solid fuel use has been associated with adverse mental health outcomes among older adults. However, evidence on the association between household solid fuel use and depressive symptoms, particularly with respect to kitchen ventilation conditions and potential geographic differences, remains limited.

**Methods:**

This cross-sectional study analyzed data from 11,697 participants aged ≥65 years in the 2018 wave of the Chinese Longitudinal Healthy Longevity Survey (CLHLS). Household fuel types and kitchen ventilation conditions were ascertained through self-reported data. Depressive symptoms were assessed using the 10-item Center for Epidemiologic Studies Depression Scale (CES-D-10). Multivariable logistic regression models and multiple linear regression models were employed to estimate associations, adjusting for a range of potential confounders.

**Results:**

Among 11,697 participants, 3,143 (26.87%) exhibited depressive symptoms, and 3,471 (29.67%) reported household solid fuel use. After adjustment for potential confounders, household solid fuel use was associated with higher odds of depressive symptoms compared with clean fuel use (OR = 1.25, 95% CI: 1.09–1.44). Similar results were observed when depressive symptoms were analyzed as a continuous CES-D-10 score (B = 0.57, 95% CI: 0.33–0.82). In exploratory analyses of specific fuel types, charcoal use showed a relatively higher odds estimate; however, this finding was based on a small number of exposed participants (*n* = 39) and should be interpreted cautiously. No significant interaction was observed between household fuel type and kitchen ventilation. Joint analysis showed that participants using solid fuels without mechanical ventilation had higher odds of depressive symptoms compared with those using clean fuels with mechanical ventilation (OR = 2.16, 95% CI: 1.63–2.86).

**Conclusion:**

Household solid fuel use was associated with depressive symptoms among older adults, with joint exposure analyses further indicating differences across fuel use and kitchen ventilation combinations. These findings highlight the potential relevance of household energy and kitchen ventilation to mental health in later life.

## Introduction

China has the world’s largest population of older adults, with people aged 65 years or older accounting for 13.50% of the total population (1, 2). As China undergoes rapid population aging, mental health problems among older adults have received increasing attention (3). Depressive symptoms are particularly common, with a prevalence of 23.6% among older adults in China (4). In addition, depressive symptoms are associated with a range of adverse outcomes, including suicidal behavior (5), cardiovascular disease (6), reduced social participation (7), cognitive decline, and poorer quality of life (8). Given the high prevalence and substantial burden, identifying potentially modifiable factors associated with depressive symptoms has important public health implications.

Epidemiological studies have reported associations between ambient air pollution and depressive symptoms among older adults (9–11). However, unlike ambient air pollution, household air pollution may expose older adults over longer periods because they spend much of their time indoors (12). Globally, approximately 2.1 billion people still rely on polluting fuels and technologies for cooking and other household energy needs, and the combustion of solid fuels, including coal, wood, crop residues, and charcoal, is a major source of household air pollution (13). Previous studies have reported associations between household solid fuel use and depressive symptoms (14–16). A longitudinal study involving 8,637 participants found that household solid fuel use was associated with a higher incidence of depressive symptoms during follow-up (14). Evidence from the China Health and Retirement Longitudinal Study further showed that solid fuel use for cooking and heating was associated with higher odds of depressive symptoms (15). A study using data from the Longitudinal Aging Study in India found that household unclean fuel use was associated with higher odds of depression among adults aged 50 years or older (16). These findings suggest that household solid fuel use is a potentially modifiable household environmental factor associated with depressive symptoms among older adults.

Fuel type alone may not fully capture actual exposure intensity, because the accumulation of indoor pollutants during cooking is also related to household ventilation conditions (17). Kitchen ventilation represents a key and modifiable determinant of indoor pollutant exposure, a and mechanical ventilation devices, such as range hoods and exhaust fans, can reduce concentrations of cooking-related particulate matter and combustion-derived pollutants (17, 18). Compared with large-scale clean energy transitions that depend on infrastructure and fuel substitution, improving kitchen ventilation represents a feasible and cost-effective household-level exposure control strategy, particularly in resource-limited rural settings where reliance on solid fuels persists (19, 20). Accordingly, considering solid fuel use together with kitchen ventilation may provide a more comprehensive characterization of household air pollution exposure than assessing solid fuel use alone. However, to our knowledge, only two studies, both based on the Henan Rural Cohort, have examined the joint association of household solid fuel use and kitchen ventilation with depressive symptoms (21, 22). Both studies reported higher odds of depressive symptoms among participants using solid fuels with natural ventilation than among those using clean fuels with mechanical ventilation. These studies were conducted in rural populations within a geographically restricted area, and evidence from large nationwide samples of older adults remains limited.

This study used data from the 2018 Chinese Longitudinal Healthy Longevity Survey (CLHLS) to examine the association between household solid fuel use and depressive symptoms among adults aged 65 years or older. Two previous studies using the same CLHLS data considered household solid fuel use in the contexts of socioeconomic status and household mold exposure, respectively (23, 24). Unlike these studies, the present study incorporated kitchen ventilation as an important household environmental factor and examined the joint association of household solid fuel use and kitchen ventilation with depressive symptoms. By jointly considering the type of fuel used for cooking and kitchen ventilation, this study characterized household exposure conditions more comprehensively. This joint assessment adds new evidence to the existing literature. We also examined whether the association between household solid fuel use and depressive symptoms differed between northern and southern China. By examining this regional variation, we aimed to provide evidence relevant to policy development in household energy, environmental health, and mental health.

## Materials and methods

### Data source and participant selection

The Chinese Longitudinal Healthy Longevity Survey (CLHLS) is a nationwide cohort study targeting adults aged 65 and older, designed to investigate the health status of older adults in China and its associated social, behavioral, and biological determinants. The survey recruited participants from approximately 630 counties and cities across 23 provinces in mainland China, covering 85% of the national population. To ensure data reliability, interviews were conducted by trained survey staff in participants’ homes using structured questionnaires, systematically collecting detailed information on sociodemographic characteristics, lifestyle behaviors, health status, psychological traits, household environmental exposures, and disease profiles. Comprehensive details regarding the study protocol and data quality assurance measures have been documented in preceding publications (25).

**Figure 1:**
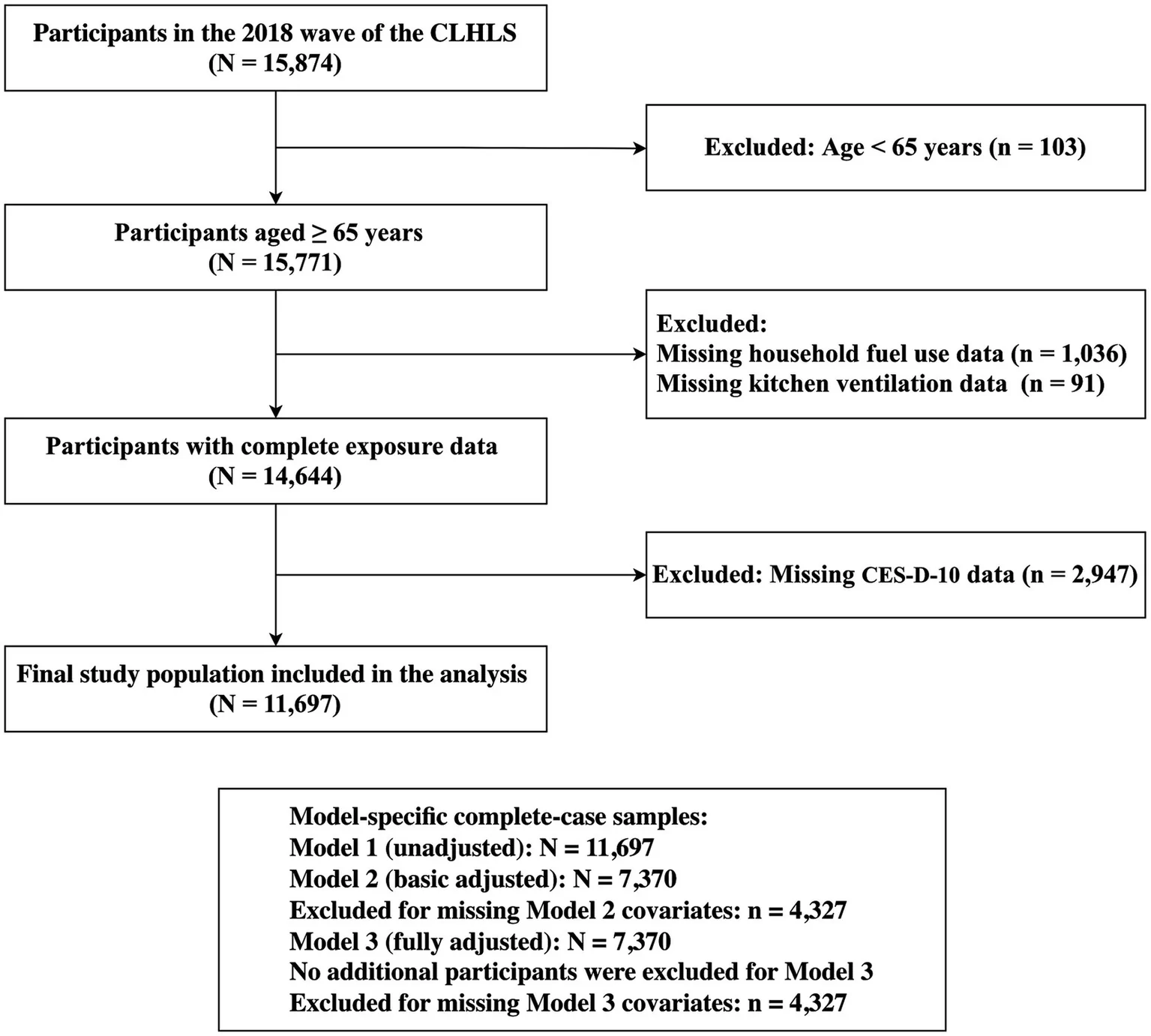
Flow chart of participant selection and analytic samples.

The present study used data from the 2018 wave of the CLHLS, which provided concurrent information on household cooking fuel type, kitchen ventilation, depressive symptoms, and relevant covariates. The study was designed to examine cross-sectional associations within this wave. Among 15,874 participants, 103 aged <65 years and those with missing information on household solid fuel use (*n* = 1,036), kitchen ventilation (*n* = 91), or depressive symptoms assessed by CES-D-10 (*n* = 2,947) were excluded. Finally, 11,697 participants with complete information on the primary exposure and outcome variables were included in the main analysis. Regression analyses were conducted using complete-case data for the variables included in each model. Model 1 included all 11,697 participants. Model 2 included 7,370 participants after excluding those with missing covariate data. Model 3 further adjusted for kitchen ventilation alongside the covariates included in Model 2, with an analytic sample of 7,370 participants. The participant selection process and the analytic sample size for each model are shown in Figure 1.

The CLHLS received ethical approval from the Biomedical Ethics Committee of Peking University (IRB00001052-13074), and written informed consent was obtained from all participants or their legal representatives. This study was based on de-identified secondary data and involved no direct interaction with participants.

### Assessment of exposure

Household cooking fuel use was assessed based on the question: “Which type of fuel is normally used for cooking in your home?” Based on reported cooking fuel types, participants were classified into two groups: clean fuel (electricity, natural gas, liquefied petroleum gas, or solar energy) and solid fuel (coal or coke, charcoal, firewood, or straw) (23, 26). Kitchen ventilation was assessed based on the question: “What is the ventilation condition of your kitchen during cooking?” Participants were categorized into three groups according to their reported ventilation conditions: mechanical ventilation (range hoods or exhaust fans), natural ventilation (windows or doors), and no ventilation.

### Measurement of outcome

Depressive symptoms were evaluated using the 10-item Center for Epidemiologic Studies Depression Scale (CES-D-10) (27). Participants rated the frequency of specific symptoms over the past week on a four-point Likert scale ranging from 0 (rarely or never) to 3 (always). After reverse-coding the two positive affect items, item responses were summed to yield a total score ranging from 0 to 30, with higher values indicating greater symptom severity. Consistent with established criteria (28, 29), a cutoff score of ≥10 was used to identify participants with clinically significant depressive symptoms. Throughout the manuscript, the outcome was uniformly referred to as screening-defined elevated depressive symptoms, rather than depression diagnosed by clinical assessment. The scale demonstrated satisfactory internal consistency in the present study, with a Cronbach’s alpha of 0.738.

### Covariates

To minimize potential confounding bias, covariates were selected based on previous literature (30–33). The included questionnaire-derived covariates were age (continuous), gender (male/female), marital status (married, never married, divorced/widowed), educational level (elementary school or below, junior high school, senior high school or above), region (north/south), residence (city/town/rural), annual household income (<30,000 yuan, 30,000–50,000 yuan, >50,000 yuan), occupation before age 60 (manual worker/non-manual worker), smoking status (non-smoker, former smoker, current smoker), drinking status (non-drinker, former drinker, current drinker), physical activity (no/yes), and self-reported chronic conditions (including hypertension, diabetes, heart disease, arthritis, and respiratory diseases; no/yes). Detailed information on the corresponding CLHLS questionnaire items, original response options, and coding or recategorization procedures is provided in Supplementary Table S1.

**Figure 2:**
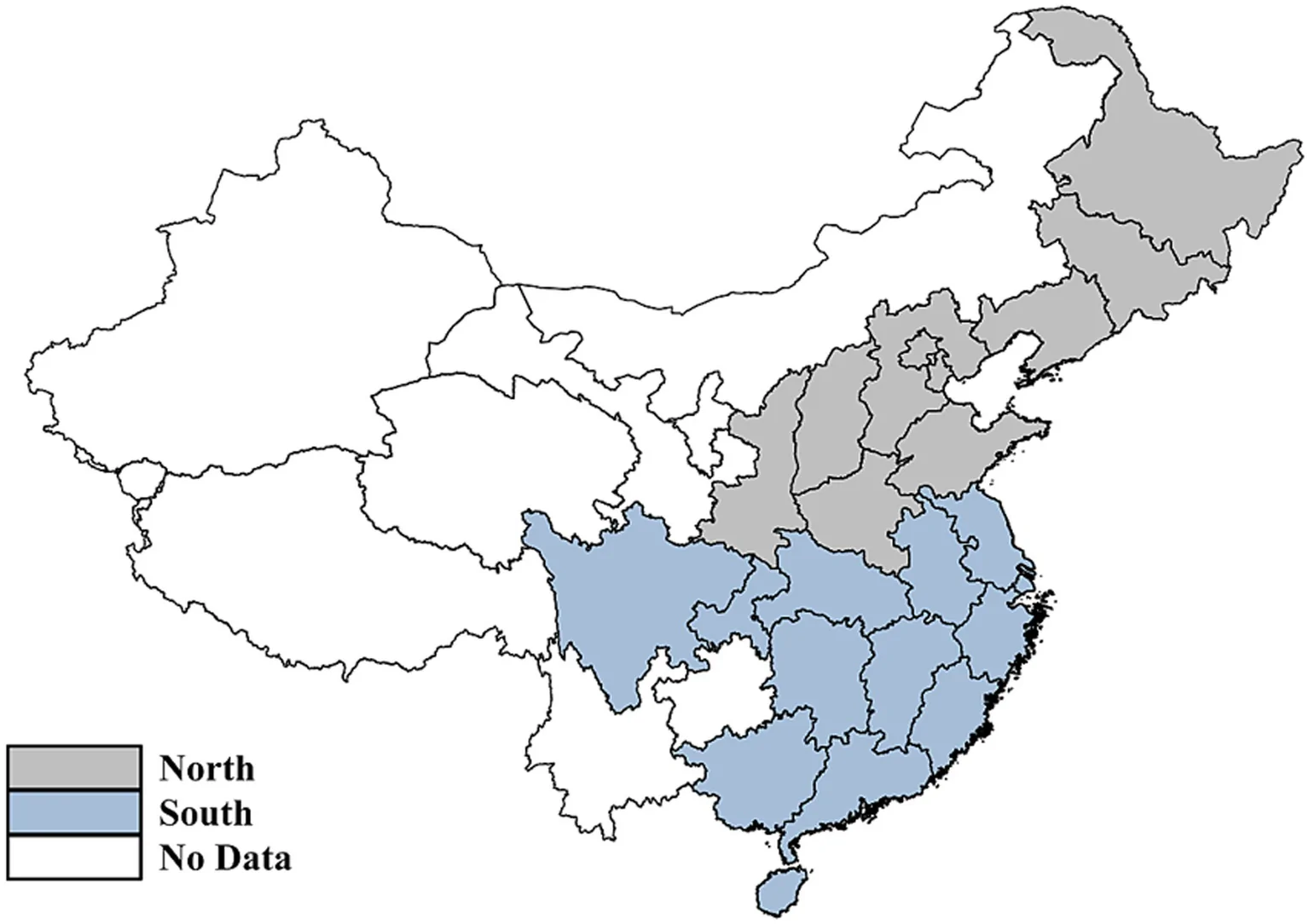
North–south geographic classification of the 23 study regions in China.

Region was classified as North or South according to participants’ provinces of residence, following the Outline of China’s Physical Geographic Regionalization (Figure 2). Annual ambient PM_2.5_ concentrations were obtained from the China regional Sat-PM_2.5_ dataset developed by the Atmospheric Composition Analysis Group (version V6. GL.02.03). This dataset integrates satellite-derived aerosol optical depth, GEOS-Chem simulations, and ground-based monitoring data to estimate ground-level PM_2.5_ concentrations. Provincial annual mean PM_2.5_ concentrations were calculated by averaging gridded estimates within each provincial boundary. Each participant was assigned the annual mean PM_2.5_ concentration corresponding to their province of residence and interview year, and PM_2.5_ was included as a continuous covariate in the regression models to adjust for potential confounding related to ambient air pollution (34).

### Statistical analysis

Descriptive statistics were used to summarize participant characteristics. Continuous variables were presented as means and standard deviations, and categorical variables were presented as frequencies and percentages. Missing values for each covariate were summarized in Supplementary Table S2. Differences in participant characteristics by depressive symptom status and household solid fuel use were examined using independent-sample t tests and chi-square tests. In addition, baseline characteristics were compared between participants included in the final analysis and those excluded due to missing CES-D-10 data.

Multivariable logistic regression models were used to examine the associations of household solid fuel use and specific fuel types (coal or coke, charcoal, and firewood/straw) with depressive symptoms. Associations were presented as odds ratios (ORs) and 95% confidence intervals (CIs). Multivariable linear regression models were additionally conducted to assess the association between household solid fuel use and continuous CES-D-10 scores, with effect estimates expressed as unstandardized regression coefficients (B) and 95% CIs. Three sequential regression models were constructed. Model 1 was unadjusted. Model 2 was adjusted for age, gender, marital status, region, residence, educational level, household annual income, occupation before age 60, smoking status, drinking status, physical activity, hypertension, diabetes, heart disease, arthritis, respiratory diseases, and PM_2.5_. Model 3 was further adjusted for kitchen ventilation in addition to the covariates included in Model 2 (21). Primary regression analyses were performed using complete-case data for all variables included in each model.

Subgroup analyses were conducted using the fully adjusted model according to age (65–79, 80–99, and ≥100 years), gender, marital status, region, residence, educational level, household annual income, occupation before age 60, physical activity, and kitchen ventilation. Interaction terms were included to assess whether the associations between household solid fuel use and depressive symptoms differed across subgroups. The fuel-specific analyses were exploratory.

To assess the joint associations of household solid fuel use and kitchen ventilation with depressive symptoms, participants were categorized into six groups according to the combination of fuel type (clean fuel or solid fuel) and ventilation status (mechanical ventilation, natural ventilation, or no ventilation). Multivariable logistic regression models were used to estimate adjusted ORs and 95% CIs for each joint exposure group, with clean fuel use combined with mechanical ventilation as the reference group.

Four sensitivity analyses were performed to evaluate the robustness of the findings. First, modified Poisson regression models with robust variance estimation were used to estimate prevalence ratios (PRs) and 95% CIs. Second, multiple imputation was performed among participants included in the primary analyses (*n* = 11,697) to assess the potential influence of missing covariate data. The imputation model included household fuel use, kitchen ventilation, depressive symptoms, continuous CES-D-10 scores, and all covariates included in the fully adjusted model. Only missing covariate values were imputed. Multiple imputation was conducted using the chained equations approach implemented in the mice package in R. Twenty imputed datasets were generated with five iterations. Predictive mean matching was used for continuous variables, logistic regression for binary variables, and logistic or multinomial regression models were applied for categorical variables according to variable type. Regression models were fitted separately in each imputed dataset, and estimates were pooled using Rubin’s rules. Complete-case analyses were considered the primary analyses, while multiple imputation analyses were conducted as sensitivity analyses. Finally, to reduce potential recall bias and improve data reliability, sensitivity analyses were performed after excluding participants with self-reported dementia and those who had moved within the previous five years. Statistical analyses were conducted using SPSS 27.0 and R 4.5.2, with a two-sided *p* value < 0.05 considered statistically significant.

## Results

### Characteristics of the study population

Table 1 outlines the baseline characteristics of the study population, comprising 11,697 participants with a mean age of 83.11 years, among whom 6,230 (53.26%) were female. The study identified 3,143 older adults with self-reported depressive symptoms, yielding a prevalence rate of 26.87%; concurrently, 3,471 (29.67%) participants reported using solid fuels for cooking. Participants with depressive symptoms were more likely to be female, older, reside in southern regions, have lower educational attainment and lower annual household income, have been classified as manual workers before age 60, and use natural ventilation (All *p* < 0.001). Regarding household fuel use, differences were observed in residential and geographic characteristics. Compared with clean fuel users, solid fuel users were significantly more likely to reside in rural areas and northern regions (All *p* < 0.001). Furthermore, solid fuel users were more likely to use natural ventilation than clean fuel users (35.12% vs. 77.33%, *p* < 0.001). Supplementary Table S3 compares the baseline characteristics of participants included in the analytical sample and those excluded because of missing CES-D-10 data. Compared with included participants, excluded participants were older, more likely to be female, less likely to be divorced/widowed, and had lower educational level, lower household annual income, and were more likely to live in rural areas and be classified as manual workers. These differences suggest that missing CES-D-10 data may not have occurred completely at random and indicate the possibility of selection bias due to outcome missingness.

**Table 1:** Main characteristics of study participants according to depressive symptoms and household fuel use (*N* = 11,697).

Characteristics	Total	Depressive symptoms		*p* valve	Household fuel use		*p* valve
		No	Yes		Clean fuels	Solid fuels	
Participants, *N*	11,697	8,554	3,143		8,226	3,471	
Age (years, mean ± SD)	83.11 ± 11.03	82.61 ± 11.07	84.48 ± 10.83	< 0.001	83.08 ± 11.06	83.19 ± 10.96	0.602
PM_2.5_ (μg/m^3^, mean ± SD)	40.08 ± 9.52	40.54 ± 9.49	38.80 ± 9.49	< 0.001	39.17 ± 9.29	42.22 ± 9.72	< 0.001
Gender, *n* (%)				< 0.001			0.046
Male	5,467 (46.74)	4,255 (49.74)	1,212 (38.56)		3,894 (47.34)	1,573 (45.32)	
Female	6,230 (53.26)	4,299 (50.26)	1,931 (61.44)		4,332 (52.66)	1,898 (54.68)	
Marital status, *n* (%)				< 0.001			< 0.001
Married	5,596 (48.31)	4,351 (51.32)	1,245 (40.11)		3,870 (47.46)	1,726 (50.35)	
Never married	77 (0.66)	44 (0.52)	33 (1.06)		45 (0.55)	32 (0.93)	
Divorced/widowed	5,910 (51.02)	4,084 (48.17)	1,826 (58.83)		4,240 (51.99)	1,670 (48.72)	
Region, *n* (%)				< 0.001			< 0.001
North	3,607 (30.84)	2,844 (33.25)	763 (24.28)		2,374 (28.86)	1,233 (35.52)	
South	8,090 (69.16)	5,710 (66.75)	2,380 (75.72)		5,852 (71.14)	2,238 (64.48)	
Residence, *n* (%)				< 0.001			< 0.001
City	2,624 (22.43)	2,075 (24.26)	549 (17.47)		2,581 (31.38)	43 (1.24)	
Town	3,858 (32.98)	2,704 (31.61)	1,154 (36.72)		2,561 (31.13)	1,297 (37.37)	
Rural	5,215 (44.58)	3,775 (44.13)	1,440 (45.82)		3,084 (37.49)	2,131 (61.39)	
Educational level (*n*, %)				< 0.001			< 0.001
Elementary school or below	7,881 (78.66)	5,620 (76.53)	2,261 (84.52)		5,218 (73.31)	2,663 (91.80)	
Junior high school	1,101 (10.99)	898 (12.23)	203 (7.59)		928 (13.04)	173 (5.96)	
Senior high school or above	1,037 (10.35)	826 (11.25)	211 (7.89)		972 (13.66)	65 (2.24)	
Household annual income, *n* (%)				< 0.001			< 0.001
< 30,000 yuan	4,946 (46.07)	3,400 (43.09)	1,546 (54.34)		2,717 (35.69)	2,229 (71.37)	
30,000–50,000 yuan	2,227 (20.75)	1,704 (21.60)	523 (18.38)		1,686 (22.15)	541 (17.32)	
> 50,000 yuan	3,562 (33.18)	2,786 (35.31)	776 (27.28)		3,209 (42.16)	353 (11.30)	
Occupation before age 60, *n* (%)				< 0.001			< 0.001
Manual worker	8,801 (88.20)	6,321 (86.46)	2,480 (92.95)		5,991 (84.31)	2,810 (97.81)	
Non-manual worker	1,178 (11.80)	990 (13.54)	188 (7.05)		1,115 (15.69)	63 (2.19)	
Smoking status, *n* (%)				< 0.001			< 0.001
Non–smoker	7,858 (68.28)	5,578 (66.31)	2,280 (73.64)		5,483 (67.82)	2,375 (69.38)	
Former smoker	1,740 (15.12)	1,352 (16.07)	388 (12.53)		1,330 (16.45)	410 (11.98)	
Current smoker	1,910 (16.60)	1,482 (17.62)	428 (13.82)		1,272 (15.73)	638 (18.64)	
Drinking status, *n* (%)				< 0.001			0.018
Non–drinker	8,285 (72.36)	5,939 (70.95)	2,346 (76.19)		5,851 (72.62)	2,434 (71.74)	
Former drinker	1,359 (11.87)	992 (11.85)	367 (11.92)		981 (12.18)	378 (11.14)	
Current drinker	1,806 (15.77)	1,440 (17.20)	366 (11.89)		1,225 (15.20)	581 (17.12)	
Physical activity, *n* (%)				< 0.001			< 0.001
No	7,534 (65.35)	5,137 (60.89)	2,397 (77.52)		4,886 (60.29)	2,648 (77.34)	
Yes	3,994 (34.65)	3,299 (39.11)	695 (22.48)		3,218 (39.71)	776 (22.66)	
Hypertension, *n* (%)				< 0.001			< 0.001
No	6,025 (55.21)	4,524 (56.21)	1,501 (52.43)		3,994 (51.88)	2,031 (63.19)	
Yes	4,887 (44.79)	3,525 (43.79)	1,362 (47.57)		3,704 (48.12)	1,183 (36.81)	
Diabetes, *n* (%)				< 0.001			< 0.001
No	9,327 (88.95)	6,962 (89.70)	2,365 (86.79)		6,449 (86.98)	2,878 (93.68)	
Yes	1,159 (11.05)	799 (10.30)	360 (13.21)		965 (13.02)	194 (6.32)	
Heart disease, *n* (%)				< 0.001			< 0.001
No	8,583 (81.56)	6,459 (83.02)	2,124 (77.41)		5,901 (79.36)	2,682 (86.85)	
Yes	1,941 (18.44)	1,321 (16.98)	620 (22.59)		1,535 (20.64)	406 (13.15)	
Arthritis, *n* (%)				< 0.001			< 0.001
No	9,222 (88.36)	6,952 (90.02)	2,270 (83.64)		6,467 (87.68)	2,755 (90.00)	
Yes	1,215 (11.64)	771 (9.98)	444 (16.36)		909 (12.32)	306 (10.00)	
Respiratory diseases, *n* (%)				< 0.001			0.022
No	9,120 (88.64)	6,836 (90.09)	2,284 (84.56)		6,406 (88.18)	2,714 (89.75)	
Yes	1,169 (11.36)	752 (9.91)	417 (15.44)		859 (11.82)	310 (10.25)	
Kitchen ventilation, *n* (%)				< 0.001			< 0.001
Mechanical ventilation	5,097 (43.58)	3,997 (46.73)	1,100 (35.00)		4,841 (58.85)	256 (7.38)	
Natural ventilation	5,573 (47.64)	3,921 (45.84)	1,652 (52.56)		2,889 (35.12)	2,684 (77.33)	
No ventilation	1,027 (8.78)	636 (7.44)	391 (12.44)		496 (6.03)	531 (15.30)	

Percentages were calculated based on available data for each covariate; therefore, denominators may vary across variables.

### Association of household solid fuel use with depressive symptoms

Table 2 presents the associations between household fuel use and depressive symptoms. In the unadjusted logistic regression model, solid fuel use was associated with higher odds of depressive symptoms compared with clean fuel use (OR = 1.51, 95% CI: 1.39–1.65, *p* < 0.001). The association remained statistically significant in the fully adjusted model (OR = 1.25, 95% CI: 1.09–1.44, *p* = 0.001). When CES-D-10 scores were treated as a continuous outcome in linear regression analyses, the results were similar to those of the logistic regression analyses. In the unadjusted model, solid fuel users had CES-D-10 scores that were 1.09 points higher than those of clean fuel users (B = 1.09, 95% CI: 0.91–1.26, *p* < 0.001). In the fully adjusted model, solid fuel users had CES-D-10 scores that were 0.57 points higher than those of clean fuel users (B = 0.57, 95% CI: 0.33–0.82, *p* < 0.001).

**Table 2:** Association between household solid fuel use and depressive symptoms (*N* = 11,697).

Model	*N*	Clean fuels	Solid fuels	P value
Depressive symptoms, OR (95% CI)				
Model 1	11,697	Reference	1.51 (1.39, 1.65)	<0.001
Model 2	7,370	Reference	1.35 (1.18, 1.54)	<0.001
Model 3	7,370	Reference	1.25 (1.09, 1.44)	0.001
CES-D-10 scores, B (95% CI)				
Model 1	11,697	Reference	1.09 (0.91, 1.26)	<0.001
Model 2	7,370	Reference	0.79 (0.55, 1.02)	<0.001
Model 3	7,370	Reference	0.57 (0.33, 0.82)	<0.001

Model 1 was unadjusted; Model 2 adjusted for age, gender, marital status, region, residence, educational level, household annual income, occupation before age 60, smoking status, drinking status, physical activity, hypertension, diabetes, heart disease, arthritis, respiratory diseases, and PM2.5; Model 3 further adjusted for kitchen ventilation based on Model 2.

### Associations of specific fuel types with depressive symptoms

Exploratory analyses by specific fuel type are shown in Table 3. In the fully adjusted model, coal or coke use (OR = 1.40, 95% CI: 1.02–1.92, *p* = 0.038), charcoal use (OR = 3.74, 95% CI: 1.48–9.50, *p* = 0.005), and firewood or straw use (OR = 1.22, 95% CI: 1.06–1.40, *p* = 0.007) were associated with higher odds of depressive symptoms compared with clean fuel use. Because only 39 participants used charcoal, this estimate was imprecise and should be interpreted as exploratory.

**Table 3:** Associations between specific fuel types and depressive symptoms (*N* = 11,697).

Variables	N _case_/N _total_	Model 1	*p* value	Model 2	*p* value	Model 3	*p* value
		OR (95% CI)		OR (95% CI)		OR (95% CI)	
Clean fuels	2,006/8,226	Reference		Reference		Reference	
Coal or coke	113/345	1.51 (1.20, 1.90)	<0.001	1.46 (1.06, 2.00)	0.020	1.40 (1.02, 1.92)	0.038
Charcoal	18/39	2.66 (1.41, 4.50)	0.002	3.92 (1.55, 9.90)	0.004	3.74 (1.48, 9.50)	0.005
Firewood or straw	1,006/3,087	1.50 (1.37, 1.64)	<0.001	1.32 (1.15, 1.51)	<0.001	1.22 (1.06, 1.40)	0.007

### Subgroup analyses and interaction analyses

**Figure 3:**
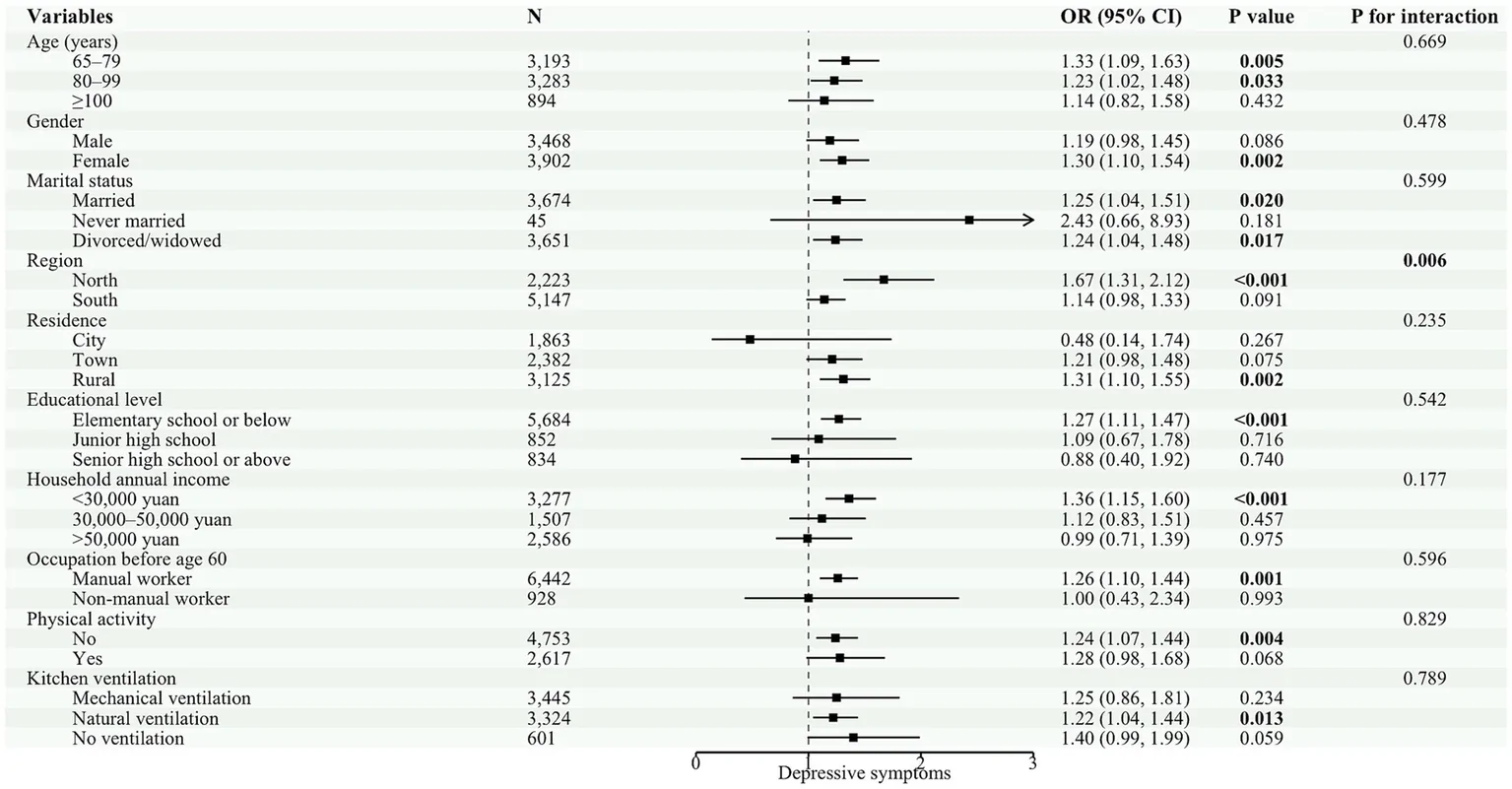
Subgroup analyses of the association between household solid fuel use and depressive symptoms. Odds ratios (*ORs*) and 95% confidence intervals (*CIs*) were estimated using multivariable logistic regression, adjusted for age, gender, marital status, region, residence, educational level, household annual income, occupation before age 60, smoking status, drinking status, physical activity, hypertension, diabetes, heart disease, arthritis, respiratory diseases, PM_2.5_, and kitchen ventilation, except for the variable defining each stratum. *P* for interaction was calculated using likelihood-ratio tests. The arrow for the never-married subgroup indicates that the upper limit of the 95% confidence interval extends beyond the displayed range of the x-axis; the complete numerical estimate is shown in the adjacent column.

Figure 3 presents the subgroup analyses conducted according to age, gender, marital status, region, residence, educational level, household annual income, occupation before age 60, physical activity, and kitchen ventilation. A significant interaction was observed between household solid fuel use and region in relation to depressive symptoms (P for interaction = 0.006). When stratified by region, household solid fuel use was associated with higher odds of depressive symptoms in northern China (OR = 1.67, 95% CI: 1.31–2.12), whereas no statistically significant association was observed in southern China (OR = 1.14, 95% CI: 0.98–1.33). No significant interactions were observed for age, gender, marital status, residence, educational level, household annual income, occupation before age 60, physical activity, or kitchen ventilation (all P for interaction > 0.05). Because multiple interaction tests were conducted without adjustment for multiple comparisons, the observed regional interaction may represent a chance finding and should be interpreted with caution.

### Joint associations of household solid fuel use and kitchen ventilation with depressive symptoms

**Figure 4:**
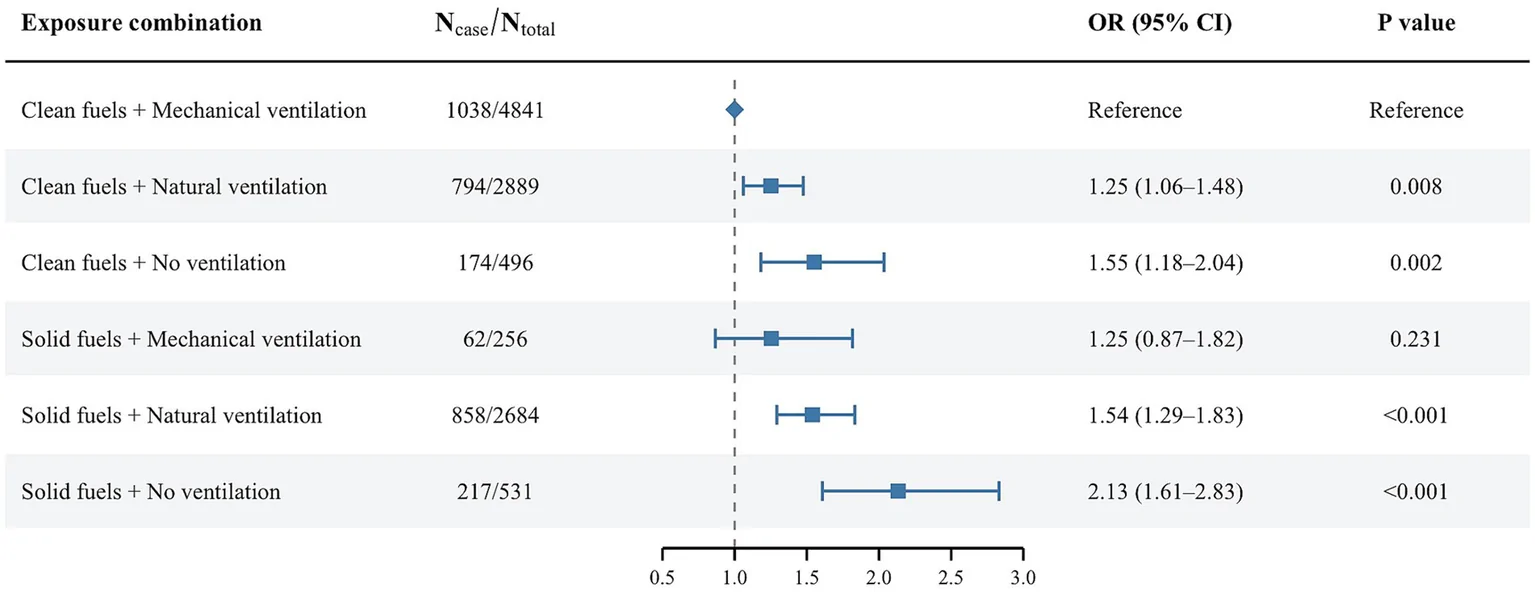
Joint analysis of household solid fuel use and kitchen ventilation on the association with depressive symptoms. Odds ratios (ORs) and 95% confidence intervals (CIs) were estimated using multivariable logistic regression, adjusted for age, gender, marital status, region, residence, educational level, household annual income, occupation before age 60, smoking status, drinking status, physical activity, hypertension, diabetes, heart disease, arthritis, respiratory diseases, and PM_2.5_.

Figure 4 summarizes the joint associations of household solid fuel use and kitchen ventilation with depressive symptoms. Compared with participants using clean fuels with mechanical ventilation, those using solid fuels without kitchen ventilation had the highest odds of depressive symptoms (OR = 2.13, 95% CI: 1.61–2.83, *p* < 0.001). The joint analysis compared each exposure combination with the reference group and should not be interpreted as evidence of interaction or effect modification.

### Sensitivity analyses

Sensitivity analyses supported the direction of the primary association (Supplementary Tables S4–S7). In the modified Poisson regression analysis, household solid fuel use was associated with a 17% higher prevalence of depressive symptoms than clean fuel use (PR = 1.17, 95% CI: 1.06–1.28, *p* < 0.001; Supplementary Table S4). In the multiple-imputation analysis based on 20 imputed datasets, in which only missing covariates were imputed, the pooled OR for depressive symptoms was 1.24 (95% CI: 1.11–1.37, p < 0.001). When the CES-D-10 score was analyzed as a continuous outcome, household solid fuel use was associated with a 0.53-point higher score than clean fuel use (pooled B = 0.53, 95% CI: 0.33–0.72, p < 0.001; Supplementary Table S5). Similar associations were observed after excluding participants with self-reported dementia (OR = 1.28, 95% CI: 1.11–1.46; B = 0.61, 95% CI: 0.36–0.85; Supplementary Table S6) and after restricting the analysis to participants who had not changed their residential address in the past five years (OR = 1.26, 95% CI: 1.10–1.45; B = 0.58, 95% CI: 0.33–0.83; Supplementary Table S7).

## Discussion

In this national cross-sectional study, household solid fuel use was associated with higher odds of depressive symptoms and higher CES-D-10 scores than clean fuel use. In exploratory analyses of specific fuel types, charcoal use showed the strongest association; however, the small number of charcoal users limits the precision of this estimate and warrants cautious interpretation. Subgroup analyses suggested that the association varied by geographic region and was stronger in northern China. However, as no adjustment was made for multiple comparisons, this finding should be interpreted with caution. In the joint analysis, the strongest association was observed among participants using solid fuels with no kitchen ventilation, compared with those using clean fuels with mechanical ventilation. However, no statistically significant interaction was identified between household fuel type and kitchen ventilation, indicating that the observed pattern reflects differences across exposure combinations rather than effect modification. Overall, these findings highlight the importance of considering household fuel use and kitchen ventilation together in relation to depressive symptoms among older adults.

Household solid fuel combustion is a major source of household air pollution. Several studies have reported associations between solid fuel use and depressive symptoms among older adults (35–37). An analysis of 13,361 older participants from the CLHLS reported that biomass fuel use for cooking was associated with 23% higher odds of depressive symptoms (35). Similarly, a large cohort study from the China Kadoorie Biobank found that longer durations of solid fuel use were associated with depressive symptoms among rural older adults (36). A cross-sectional study conducted in six low- and middle-income countries also reported that polluting cooking fuel use was associated with 57% higher odds of depressive symptoms among adults aged 50 years and older, with stronger associations among rural residents and women (37). In summary, the above studies support the association between household solid fuel use and depressive symptoms among older adults. This is consistent with the main finding of our study. Several biological pathways may help explain the observed association between household solid fuel use and depressive symptoms among older adults. Solid fuel combustion releases particulate matter (PM_2.5_, PM_10_), carbon monoxide (CO), and other gaseous pollutants, which cause substantially to household air pollution (38, 39). Solid fuel use may involve long-term exposure to PM_2.5_, which is associated with systemic oxidative stress and chronic low-grade inflammation and plays an important role in neurodegenerative diseases (40–43). Studies have further suggested that neurodegenerative diseases are also linked to the pathophysiology of depressive symptoms (44). Together, these findings provide a biologically plausible explanation for the observed association between household solid fuel use and depressive symptoms. In addition, respiratory and cardiovascular dysfunction associated with particulate exposure may provide further explanations through lung–brain and heart–brain interactions and chronic cerebral hypoxia (45–47). In the exploratory fuel-specific analysis, charcoal use showed the most pronounced association with depressive symptoms. Previous observational studies have linked carbon monoxide poisoning from charcoal combustion to neurological and psychiatric manifestations, providing limited biological plausibility for this finding (48, 49). Nevertheless, it should be noted that the proportion of participants exposed to charcoal in our study was relatively small, which may limit statistical precision and increase uncertainty in effect estimates. Therefore, these results should be interpreted with caution and confirmed in larger prospective studies.

Social and structural factors may also help explain the observed association. Solid fuel use is often concentrated in households with fewer socioeconomic resources, and previous research among older adults in China has shown that socioeconomic status is closely related to both household fuel use and depressive symptoms (23). Thus, solid fuel use may partly reflect underlying socioeconomic disadvantage rather than household air-pollution exposure alone. The residential environment may offer another perspective on the observed association. A recent study of middle-aged and older adults in China found that the residential environment partly mediated the association between cooking with solid fuels and depressive symptoms, suggesting that housing and living conditions may partly help explain the association between household solid fuel use and depressive symptoms (50). Our findings should also be interpreted in the context of energy poverty and social isolation. Energy poverty has been associated with poorer mental health, suggesting that difficulties in meeting basic household energy needs could be associated with depressive symptoms (51). Social isolation represents another important social context. Zhao et al. reported that household solid fuel use and social isolation were jointly associated with depressive symptoms, with the strongest association observed in the group characterized by both solid fuel use and social isolation (52). Taken together, the association observed in our study may reflect not only household air-pollution exposure but also the socioeconomic, residential, and social circumstances in which solid fuels are used. Although our analyses adjusted for several socioeconomic characteristics, housing quality, energy poverty, and social isolation were not fully captured in the CLHLS, and residual confounding remains possible.

Regarding demographic and socioeconomic characteristics, stratified analyses showed statistically significant associations among women, rural residents, participants with elementary education or below, and those with an annual household income below 30,000 yuan. The finding that household solid fuel use was significantly associated with depressive symptoms among women is consistent with previous studies (29, 37, 53). In many Chinese households, women are primarily responsible for cooking and family care and may therefore experience more frequent exposure to pollutants released during solid fuel combustion (54). The associations observed among participants with lower educational attainment and lower household income may reflect socioeconomic constraints, including limited access to cleaner household energy and adequate ventilation, which may be associated with both greater household air pollution exposure and depressive symptoms (11, 23). In addition, the association observed among rural residents may be attributed to poorer household ventilation and lower-quality fuels, which may increase exposure to toxic pollutants (55). These findings highlight the potential value of promoting cleaner cooking fuels and improving household ventilation in rural and socioeconomically disadvantaged households. However, these subgroup findings do not provide evidence of interaction and should be interpreted cautiously. Future prospective studies with larger sample sizes are needed to confirm these findings.

In interaction analyses, a statistically significant interaction was observed between household cooking fuel type and geographic region. Solid fuel use was associated with depressive symptoms in northern China, whereas no statistically significant association was observed in southern China. Although previous studies have reported associations between solid fuel use and depressive symptoms in specific regions of China (56), north–south heterogeneity within China has received limited attention. The reasons underlying the observed regional difference remain unclear. Regional variation in residential ventilation and indoor exposure conditions may nevertheless provide relevant context for the association observed in northern China. Studies conducted in northeastern China have reported seasonal differences in window-opening behavior and indoor pollutant concentrations, as well as limited air exchange and elevated pollutant levels in residential kitchens during cold weather (55). However, these factors were not directly assessed in the present study and therefore cannot be used to explain the observed north–south difference. Cooking fuel type is only a proxy for household air pollution and does not capture stove type, fuel stacking, heating fuel, cooking duration, season, ventilation-use behavior, or measured pollutant concentrations. Therefore, the regional finding should be interpreted cautiously and confirmed in studies with more detailed exposure assessment.

In the joint analysis, participants who used solid fuels with no kitchen ventilation had the highest odds of depressive symptoms compared with those who used clean fuels with mechanical ventilation. Because the formal interaction test between cooking fuel type and kitchen ventilation was not statistically significant, this pattern should be interpreted as differences across joint exposure categories rather than evidence of interaction. Previous studies have reported that kitchen ventilation is associated with lower indoor concentrations of pollutants generated during solid fuel combustion, and that the use of exhaust fans or range hoods is associated with lower internal exposure to polycyclic aromatic hydrocarbons (17, 57). These findings provide a plausible context for the pattern observed across the different fuel–ventilation combinations. However, because household fuel use and kitchen ventilation were assessed categorically and indoor pollutant concentrations were not directly measured, the findings should be interpreted with caution.

To our knowledge, this study is the first to examine, in a large, nationally representative sample of older Chinese adults, the joint associations of household fuel use and kitchen ventilation with depressive symptoms. It also explored geographic variation in the association between solid fuel use and depressive symptoms and identified a statistically significant interaction by north–south region. By considering household ventilation and geographic context together, our findings extend the existing evidence on household solid fuel use and depressive symptoms.

This study has several limitations that warrant acknowledgment. First, the cross-sectional design precludes conclusions regarding temporality or causality. Future studies using multi-wave longitudinal data are needed to clarify the temporal sequence and prospective association between household solid fuel use and depressive symptoms. Second, depressive symptoms were assessed using the CES-D-10 rather than a clinical diagnosis, and cooking fuel type was self-reported, which may have introduced recall or exposure misclassification. Moreover, cooking fuel type served only as a proxy for household air pollution and did not capture stove type, fuel stacking, heating fuel, cooking duration, season, ventilation-use behavior, or measured pollutant concentrations. This limitation is particularly relevant to the regional analyses and constrains interpretation of the observed north–south differences. Finally, the inclusion of only older Chinese adults may limit the generalizability of our findings to populations with different ethnic, cultural, environmental, and socioeconomic backgrounds. Despite adjustment for a broad range of covariates, residual confounding from unmeasured or insufficiently measured factors cannot be entirely excluded.

## Conclusion

In this large national cross-sectional study of older adults in China, household solid fuel use was associated with higher odds of depressive symptoms and higher CES-D-10 scores. In the joint exposure analysis, participants using solid fuels with no kitchen ventilation had the highest odds of depressive symptoms compared with those using clean fuels with mechanical ventilation; however, the formal interaction between fuel type and kitchen ventilation was not statistically significant. Exploratory interaction analyses indicated regional variation in the association, with a significant association observed in northern China. These findings support consideration of cleaner household energy and mechanical kitchen ventilation in healthy aging strategies. Future longitudinal studies with more detailed exposure assessment are warranted to confirm these findings and further clarify the observed associations.

## Data Availability

The datasets presented in this study can be found in online repositories. The names of the repository/repositories and accession number(s) can be found: the data used in this study are available from a public repository, and additional information on the CLHLS is accessible through online resources (https://opendata.pku.edu.cn/dataverse/CHADS).
