# Supplementary material for: Associations between household solid fuel use, kitchen ventilation, and depressive symptoms among older adults in China: a national cross-sectional study
Source: Front Public Health. 2026 Sep 1;14:1920233. doi: 10.3389/fpubh.2026.1920233 (PMC13574938; doi:10.3389/fpubh.2026.1920233)
Supplement: Supplementary file 1 [file Table_1.docx]

***Supplementary Material***

**Supplementary Table S1.** Definitions and coding of covariates used in the present study.

| **Variables** | **Questions in CLHLS questionnaire** | **Options in the questionnaire** | **Coding in the present study** |
| --- | --- | --- | --- |
| Age | Validated age |  | • Age: years |
| Gender | Sex | • male  • female | • Male  • Female |
| Marital status | Current marital status? | • married  • divorced  • widowed  • never married  • don't know  • missing | • Married  • Never married  • Divorced/widowed: divorced, widowed  • missing: don't know, missing |
| Region | Province | • Beijing  • Tianjin  • Hebei  • Shanxi  • Liaoning  • Jilin  • Heilongjiang  • Shanghai  • Jiangsu  • Zhejiang  • Anhui  • Fujian  • Jiangxi  • Shandong  • Henan  • Hubei  • Hunan  • Guangdong  • Guangxi  • Hainan  • Chongqing  • Sichuan  • Shaanxi | • North: Beijing, Tianjin, Hebei, Shanxi, Liaoning, Jilin, Heilongjiang, Shandong, Henan, Shaanxi  • South: Shanghai, Jiangsu, Zhejiang, Anhui, Fujian, Jiangxi, Hubei, Hunan, Guangdong, Guangxi, Hainan, Chongqing, Sichuan |
| Residence | Current residence area of interviewee? | • city  • town  • rural | • City  • Town  • Rural |
| Educational level | How many years did you attend school? | • years of school  • don't know  • missing | • Elementary school or below = 0-6  • Junior high school = 7-9  • Senior high school or above ≥ 10  • missing: don't know, missing |
| Household annual income | What was your household’s total income last year? | • total income  • more than 100,000  • don't know  • missing | • < 30,000 yuan: 0-29,900  • 30,000–50,000 yuan: 30,000-50,000  • > 50,000 yuan: ≥ 50,100, more than 100,000  • missing: don't know, missing |
| Occupation before age 60 | What was your occupation before the age of 60? | • professional and technical personnel  • governmental, institutional or managerial personnel  • commercial, service or industrial worker  • self-employed  • agriculture, forestry, animal husbandry or fishery worker  • houseworker  • military personnel  • never worked  • others  • missing | Manual worker: commercial, service or industrial worker; self-employed; agriculture, forestry, animal husbandry or fishery worker; houseworker; military personnel; never worked; others  Non-manual worker: professional and technical personnel; governmental, institutional or managerial personnel  • missing |
| Smoking status | • Do you smoke at the present time?  • Did you smoke in the past? | • yes  • no  • don't know  • missing | • Non–smoker:  Do you smoke at the present time? = no; Did you smoke in the past? = no  • Former smoker:  Do you smoke at the present time? = no; Did you smoke in the past? = yes  • Current smoker:  Do you smoke at the present time? = yes; Did you smoke in the past? = yes/no  • missing: don't know, missing |
| Drinking status | • Do you drink alcohol at the present time?  • Did you drink alcohol in the past? | • yes  • no  • don't know  • missing | • Non–drinker:  Do you drink alcohol at the present time? = no; Did you drink alcohol in the past? = no  • Former drinker:  Do you drink alcohol at the present time? = no; Did you drink alcohol in the past? = yes  • Current drinker:  Do you drink alcohol at the present time? = yes; Did you drink alcohol in the past? = yes/no  • missing: don't know, missing |
| Physical activity | Do you do exercises regularly at present? | • yes  • no  • don't know  • missing | • Yes: yes  • No: no  • missing: don't know, missing |
| Hypertension | Are you suffering from this disease? | • yes  • no  • don't know  • missing | • Yes: yes  • No: no  • missing: don't know, missing |
| Diabetes | Are you suffering from this disease? | • yes  • no  • don't know  • missing | • Yes: yes  • No: no  • missing: don't know, missing |
| Heart disease | Are you suffering from this disease? | • yes  • no  • don't know  • missing | • Yes: yes  • No: no  • missing: don't know, missing |
| Arthritis | Are you suffering from this disease? | • yes  • no  • don't know  • missing | • Yes: yes  • No: no  • missing: don't know, missing |
| Respiratory diseases | Are you suffering from this disease? | • yes  • no  • don't know  • missing | • Yes: yes  • No: no  • missing: don't know, missing |

More detailed information about these covariates can be found on: <https://agingcenter.duke.edu/CLHLS>. Abbreviations: CLHLS = Chinese Longitudinal Healthy Longevity Survey

**Supplementary Table S2.** Number (percent) of participants with missing data for each covariate (N = 11,697).

| **Variables** | **Missing data, n (%)** |
| --- | --- |
| Age | 0 (0.00) |
| PM_2.5_ | 0 (0.00) |
| Gender | 0 (0.00) |
| Marital status | 114 (0.97) |
| Region | 0 (0.00) |
| Residence | 0 (0.00) |
| Educational level | 1,678 (14.35) |
| Household annual income | 962 (8.22) |
| Occupation before age 60 | 1,718 (14.69) |
| Smoking status | 189 (1.62) |
| Drinking status | 247 (2.11) |
| Physical activity | 169 (1.44) |
| Hypertension | 785 (6.71) |
| Diabetes | 1,211 (10.35) |
| Heart disease | 1,173 (10.03) |
| Arthritis | 1,260 (10.77) |
| Respiratory diseases | 1,408 (12.04) |
| Kitchen ventilation | 0 (0.00) |

**Supplementary Table S3.** Comparison of baseline characteristics between participants included in the analytical sample and those excluded due to missing CES-D-10 data.

| Characteristics | Included (N = 11,697) | Excluded (N = 2,947) | P valve |
| --- | --- | --- | --- |
| Participants, N | 11, 697 | 2,947 |  |
| Age (years, mean ± SD) | 83.11 ± 11.03 | 93.89 ± 9.85 | < 0.001 |
| PM_2.5_ (μg/m^3^, mean ± SD) | 40.08 ± 9.52 | 40.73 ± 10.02 | 0.001 |
| Gender, n (%) |  |  | < 0.001 |
| Male | 5,467 (46.74) | 949 (32.20) |  |
| Female | 6,230 (53.26) | 1,998 (67.80) |  |
| Marital status, n (%) |  |  | < 0.001 |
| Married | 5,596 (48.31) | 569 (19.58) |  |
| Never married | 77 (0.66) | 20 (0.69) |  |
| Divorced/widowed | 5,910 (51.02) | 2,317 (79.3) |  |
| Region, n (%) |  |  | 0.124 |
| North | 3,607 (30.84) | 952 (32.30) |  |
| South | 8,090 (69.16) | 1,995 (67.70) |  |
| Residence, n (%) |  |  | < 0.001 |
| City | 2,624 (22.43) | 504 (17.10) |  |
| Town | 3,858 (32.98) | 983 (33.36) |  |
| Rural | 5,215 (44.58) | 1,460 (49.54) |  |
| Educational level (n, %) |  |  | < 0.001 |
| Elementary school or below | 7,881 (78.66) | 2,366 (93.78) |  |
| Junior high school | 1,101 (10.99) | 66 (2.62) |  |
| Senior high school or above | 1,037 (10.35) | 826 (11.25) |  |
| Household annual income, n (%) |  |  | < 0.001 |
| < 30,000 yuan | 4,946 (46.07) | 1,288 (49.56) |  |
| 30,000–50,000 yuan | 2,227 (20.75) | 578 (22.24) |  |
| > 50,000 yuan | 3,562 (33.18) | 733 (28.20) |  |
| Occupation before age 60, n (%) |  |  | < 0.001 |
| Manual worker | 8,801 (88.20) | 2,397 (94.97) |  |
| Non-manual worker | 1,178 (11.80) | 127 (5.03) |  |
| Smoking status, n (%) |  |  | < 0.001 |
| Non–smoker | 7,858 (68.28) | 2,243 (77.59) |  |
| Former smoker | 1,740 (15.12) | 379 (13.11) |  |
| Current smoker | 1,910 (16.60) | 269 (9.30) |  |
| Drinking status, n (%) |  |  | < 0.001 |
| Non–drinker | 8,285 (72.36) | 2,302 (80.07) |  |
| Former drinker | 1,359 (11.87) | 305 (10.61) |  |
| Current drinker | 1,806 (15.77) | 268 (9.32) |  |
| Physical activity, n (%) |  |  | < 0.001 |
| No | 7,534 (65.35) | 2,512 (87.31) |  |
| Yes | 3,994 (34.65) | 365 (12.69) |  |
| Hypertension, n (%) |  |  | < 0.001 |
| No | 6,025 (55.21) | 1,736 (66.01) |  |
| Yes | 4,887 (44.79) | 894 (33.99) |  |
| Diabetes, n (%) |  |  | < 0.001 |
| No | 9,327 (88.95) | 2,410 (94.55) |  |
| Yes | 1,159 (11.05) | 139 (5.45) |  |
| Heart disease, n (%) |  |  | < 0.001 |
| No | 8,583 (81.56) | 2,221 (86.35) |  |
| Yes | 1,941 (18.44) | 351 (13.65) |  |
| Arthritis, n (%) |  |  | 0.043 |
| No | 9,222 (88.36) | 2,290 (89.80) |  |
| Yes | 1,215 (11.64) | 260 (10.20) |  |
| Respiratory diseases, n (%) |  |  | 0.509 |
| No | 9,120 (88.64) | 2,238 (89.13) |  |
| Yes | 1,169 (11.36) | 273 (10.87) |  |
| Household fuel use |  |  | < 0.001 |
| Clean fuels | 8,226 (70.33) | 1,958 (66.44) |  |
| Solid fuels | 3,471 (29.67) | 989 (33.56) |  |
| Kitchen ventilation, n (%) |  |  | < 0.001 |
| Mechanical ventilation | 5,097 (43.58) | 1,144 (38.82) |  |
| Natural ventilation | 5,573 (47.64) | 1,515 (51.41) |  |
| No ventilation | 1,027 (8.78) | 288 (9.77) |  |

Abbreviations: Percentages were calculated based on available data for each covariate; therefore, denominators may vary across variables.

**Supplementary Table S4.** Modified Poisson regression sensitivity analysis of the association between household solid fuel use and depressive symptoms (N = 11,697).

| **Model** | **N** | **Clean fuels** | **Solid fuels** | ***P* value** |
| --- | --- | --- | --- | --- |
| Depressive symptoms, PR (95% CI) | | | | |
| Model 1 | 11,697 | Reference | 1.34 (1.26, 1.43) | <0.001 |
| Model 2 | 7,370 | Reference | 1.23 (1.12, 1.34) | <0.001 |
| Model 3 | 7,370 | Reference | 1.17 (1.06, 1.28) | <0.001 |

Abbreviations: PR, prevalence ratio; CI, confidence interval. Prevalence ratios and 95% confidence intervals were estimated using modified Poisson regression with robust standard errors. Model 1 was unadjusted; Model 2 adjusted for age, gender, marital status, region, residence, educational level, household annual income, occupation before age 60, smoking status, drinking status, physical activity, hypertension, diabetes, heart disease, arthritis, respiratory diseases, and PM_2.5_; Model 3 further adjusted for kitchen ventilation based on Model 2.

**Supplementary Table S5.** Sensitivity analysis of the association between household solid fuel use and depressive symptoms after multiple imputation of missing covariate data (N = 11,697).

| **Model** | **N** | **Clean fuels** | **Solid fuels** | ***P* value** |
| --- | --- | --- | --- | --- |
| Depressive symptoms, OR (95% CI) | | | | |
| Model 1 | 11,697 | Reference | 1.51 (1.39, 1.65) | <0.001 |
| Model 2 | 11,697 | Reference | 1.35 (1.23, 1.49) | <0.001 |
| Model 3 | 11,697 | Reference | 1.24 (1.11, 1.37) | <0.001 |
| CES-D-10 scores, B (95% CI) | | | | |
| Model 1 | 11,697 | Reference | 1.09 (0.91, 1.26) | <0.001 |
| Model 2 | 11,697 | Reference | 0.78 (0.60, 0.97) | <0.001 |
| Model 3 | 11,697 | Reference | 0.53 (0.33, 0.72) | <0.001 |

Abbreviations: Missing covariate data were handled using multiple imputation. Regression estimates across imputed datasets were pooled using Rubin’s rules.

Model 1 was unadjusted; Model 2 adjusted for age, gender, marital status, region, residence, educational level, household annual income, occupation before age 60, smoking status, drinking status, physical activity, hypertension, diabetes, heart disease, arthritis, respiratory diseases, and PM_2.5_; Model 3 further adjusted for kitchen ventilation based on Model 2.

**Supplementary Table S6.** Sensitivity analysis of the association between household solid fuel use and depressive symptoms after excluding participants with self-reported dementia (N = 10,314).

| **Model** | **N** | **Clean fuels** | **Solid fuels** | ***P* value** |
| --- | --- | --- | --- | --- |
| Depressive symptoms, OR (95% CI) | | | | |
| Model 1 | 10,314 | Reference | 1.49 (1.36, 1.64) | <0.001 |
| Model 2 | 7,304 | Reference | 1.37 (1.20, 1.56) | <0.001 |
| Model 3 | 7,304 | Reference | 1.28 (1.11, 1.46) | <0.001 |
| CES-D-10 scores, B (95% CI) | | | | |
| Model 1 | 10,314 | Reference | 1.00 (0.82, 1.19) | <0.001 |
| Model 2 | 7,304 | Reference | 0.82 (0.58, 1.05) | <0.001 |
| Model 3 | 7,304 | Reference | 0.61 (0.36, 0.85) | <0.001 |

Model 1 was unadjusted; Model 2 adjusted for age, gender, marital status, region, residence, educational level, household annual income, occupation before age 60, smoking status, drinking status, physical activity, hypertension, diabetes, heart disease, arthritis, respiratory diseases, and PM_2.5_; Model 3 further adjusted for kitchen ventilation based on Model 2.

**Supplementary Table S7.** Sensitivity analysis of the association between household solid fuel use and depressive symptoms after excluding participants who changed their residential address in the past five years (N = 11,185).

| **Model** | **N** | **Clean fuels** | **Solid fuels** | ***P* value** |
| --- | --- | --- | --- | --- |
| Depressive symptoms, OR (95% CI) | | | | |
| Model 1 | 11,185 | Reference | 1.51 (1.38, 1.65) | <0.001 |
| Model 2 | 7,100 | Reference | 1.36 (1.19, 1.55) | <0.001 |
| Model 3 | 7,100 | Reference | 1.26 (1.10, 1.45) | <0.001 |
| CES-D-10 scores, B (95% CI) | | | | |
| Model 1 | 11,697 | Reference | 1.07 (0.89, 1.25) | <0.001 |
| Model 2 | 7,100 | Reference | 0.79 (0.55, 1.03) | <0.001 |
| Model 3 | 7,100 | Reference | 0.58 (0.33, 0.83) | <0.001 |

Model 1 was unadjusted; Model 2 adjusted for age, gender, marital status, region, residence, educational level, household annual income, occupation before age 60, smoking status, drinking status, physical activity, hypertension, diabetes, heart disease, arthritis, respiratory diseases, and PM_2.5_; Model 3 further adjusted for kitchen ventilation based on Model 2.
